# Kinome-Wide Profiling Identifies Human WNK3 as a Target of Cajanin Stilbene Acid from *Cajanus cajan* (L.) Millsp.

**DOI:** 10.3390/ijms23031506

**Published:** 2022-01-28

**Authors:** Nadire Özenver, Onat Kadioglu, Yujie Fu, Thomas Efferth

**Affiliations:** 1 Department of Pharmacognosy, Faculty of Pharmacy, Hacettepe University, Ankara 06100, Turkey; nadire@hacettepe.edu.tr; 2 Department of Pharmaceutical Biology, Institute of Pharmaceutical and Biomedical Sciences, Johannes Gutenberg University, 55128 Mainz, Germany; kadioglu@uni-mainz.de; 3 The College of Forestry, Beijing Forestry University, Beijing 100083, China; yujie_fu@163.com

**Keywords:** cancer, food crop, mode-of-action, natural products, nutrition, targeted therapy

## Abstract

Pigeon Pea (*Cajanus cajan* (L.) Millsp.) is a common food crop used in many parts of the world for nutritional purposes. One of its chemical constituents is cajanin stilbene acid (CSA), which exerts anticancer activity in vitro and in vivo. In an effort to identify molecular targets of CSA, we performed a kinome-wide approach based on the measurement of the enzymatic activities of 252 human kinases. The serine-threonine kinase WNK3 (also known as protein kinase lysine-deficient 3) was identified as the most promising target of CSA with the strongest enzymatic activity inhibition in vitro and the highest binding affinity in molecular docking in silico. The lowest binding affinity and the predicted binding constant pKi of CSA (−9.65 kcal/mol and 0.084 µM) were comparable or even better than those of the known WNK3 inhibitor PP-121 (−9.42 kcal/mol and 0.123 µM). The statistically significant association between WNK3 mRNA expression and cellular responsiveness to several clinically established anticancer drugs in a panel of 60 tumor cell lines and the prognostic value of WNK3 mRNA expression in sarcoma biopsies for the survival time of 230 patients can be taken as clues that CSA-based inhibition of WNK3 may improve treatment outcomes of cancer patients and that CSA may serve as a valuable supplement to the currently used combination therapy protocols in oncology.

## 1. Introduction

Kinases are essential enzymes for the transmission of information in living organisms. Among the vast majority of kinases, the eukaryotic protein kinase (EPK) family covers one of the biggest superfamilies in the human genome, with a conserved catalytic domain in their structure and fundamental roles in both biological processes and diseases [1,2]. Protein kinases (PKs) transform proteins by phosphorylation, causing a functional alteration of the target protein. EPKs may phosphorylate either serine/threonine or tyrosine residues or both at the same time and are clustered based on sequence similarities, evolutionary conservation, their functions, and structural properties (e.g., outside of the catalytic domains) [1,2,3]. They regulate signal transduction and operate cellular processes, such as apoptosis, cell cycle transition, cell movement, differentiation, metabolism, and transcription. EPKs ensure intercellular communication during cell growth, the maintenance of nervous and immune system performances, homeostasis, and physiological responses through protein phosphorylation [4,5,6,7]. On the other hand, mutations and dysregulations of PKs are involved in numerous human diseases, leading to the development of pharmacological PK activators or inhibitors. The association of kinase-dependent pathways with the emergence of various diseases, including Parkinson’s disease, Alzheimer’s disease, inflammatory responses, cardiac hypertrophy, hypertension, kidney diseases, and cancer was reported [7,8,9,10].

PKs came into the spotlight as drug targets against various diseases throughout the past years. In addition to uncountable physiological processes, PKs participate in numerous pathological processes, such as cancer, inflammation, autoimmune diseases, heart disease, and infectious diseases (e.g., by *Leishmania major*, *Trypanosoma brucei*, or *T. cruzi*) [6,11]. Janus kinases (JAK), mitogen-activated protein kinases (MAPK), and spleen tyrosine kinases (SYK) were discovered as targets to treat inflammatory-relevant conditions [12,13,14]. The function of Rho-kinases (ROCKs) in the pathogenesis of cardiovascular disease sets forth their importance as potential therapeutic targets [15].

Among PK-related diseases, cancer takes an important position, due to the overexpression or dysregulation of PKs in a majority of malignancies. To mention but a few, the epidermal growth factor receptor (EGFR) is overexpressed or mutated in bladder [16], breast [17], colorectal [18], non-small-cell lung [19,20], and pancreatic cancer [21]. AKT2 (RAC-beta serine/threonine-protein kinase) is overexpressed in ovarian cancer [22] and pancreatic carcinoma [23]. Similarly, ERBB2 (Receptor tyrosine-protein kinase erbB-2) is upregulated in breast [24], esophageal [25], gastric [25], and ovarian cancer [26]. Many different PKs are involved in tumorigenesis by affecting major signaling pathways. The metastatic spread of cancer is mainly mediated by dysregulated signal transduction pathways arising from the impaired interaction between cancerous cells, surrounding non-tumorigenic cells, and the extracellular matrix [27]. Amplification, mutation, or gene fusion of tyrosine kinases (TKs) constitutively hyperactivate the PI3K (phosphatidylinositol 3-kinase)/AKT (protein Kinase B) and RAS/extracellular signal-regulated kinase (ERK) pathways in many cancers [27,28,29], providing excellent opportunities for targeted cancer therapy.

It has been estimated that one-fifth to one-third of all targets examined in the pharmaceutical industry are PKs [30]. Approximately 175 orally efficient PK inhibitors have been enrolled in clinical trials so far [31]. The Food and Drug Administration (FDA) has approved 52 therapeutics targeting almost 20 assorted PKs. The other drugs targeting another 15–20 protein kinases have been under further clinical investigation [30,31,32]. PKs interact with hydroxy groups to phosphorylate target substrates, such as TKs or serine/threonine kinases (STKs). Moreover, dual-specificity kinases, with the capability to phosphorylate either threonine or tyrosine residues, also exist [30]. For instance, the TK inhibitor sorafenib represses various kinases, e.g., vascular endothelial growth factors (VEGFs), platelet-derived growth factor receptor (PDGFR), FMS-related tyrosine kinase/FLK2/STK-2 receptor (FLT3R), murine sarcoma viral oncogene homolog B (BRAF), rearranged during transfection (RET). Of the 52 small molecule kinase inhibitors (SMKIs) recently approved by the FDA, TK inhibitors cover the majority, succeeded by STK inhibitors, and lipokinase inhibitors [30]. Despite their extended clinical use, SMKIs exhibit unwanted side effects, such as gastrointestinal toxicity, hepatotoxicity, and cardiotoxicity [33,34].

Due to their virtually inexhaustible chemical diversity, natural products represent an indispensable source of potential kinase inhibitors. As surveyed by the National Cancer Institute (NCI), USA, only one-third of all anticancer drugs approved from 1981 to 2019 were purely synthetic compounds. The rest covers unaltered natural products, botanical drugs, natural product derivatives, synthetic mimics of natural products, or synthetic compounds with a natural product pharmacophore, providing overwhelming evidence of the importance of natural sources for cancer therapy [35]. Many studies reported the kinase inhibitory properties of natural products. Numerous natural products were shown to inhibit various kinase targets, such as VEGF, epidermal growth factor receptor (EGFR), Janus kinase (JAK), tyrosine-protein kinase ABL1 (ABL), insulin-like growth factor 1 receptor (IGF-1R), platelet-derived growth factor receptors (PDGFR), the casein kinase 1 family (CK1), protein kinase C (PKC), or the calcium/calmodulin-dependent kinase family (CAMK) [36,37]. Natural products usually display their effects through multiple targets. However, such features are not regarded as non-specificity but as multi-targeted specificity [38]. Natural products specifically addressing several targets at the same time open opportunities to fight the development of drug resistance, e.g., in cases where point mutations in one kinase render resistance to mono-specific drugs.

Cajanin stilbene acid (CSA) is a natural stilbene obtained from Pigeon Pea (*Cajanus cajan* (L.) Millsp.), which is used as a food crop in many regions worldwide. Its antitumor properties were demonstrated in vitro and in vivo [39,40,41]. However, the exact mechanisms of action and the specific targets have not been fully elucidated. In the present paper, we profile the inhibitory activity of CSA towards a large array of 252 human kinases to identify new targets associated with CSA’s cytotoxicity towards cancer cells.

## 2. Results

### 2.1. Kinase Activity Profiling

**Figure 1:**
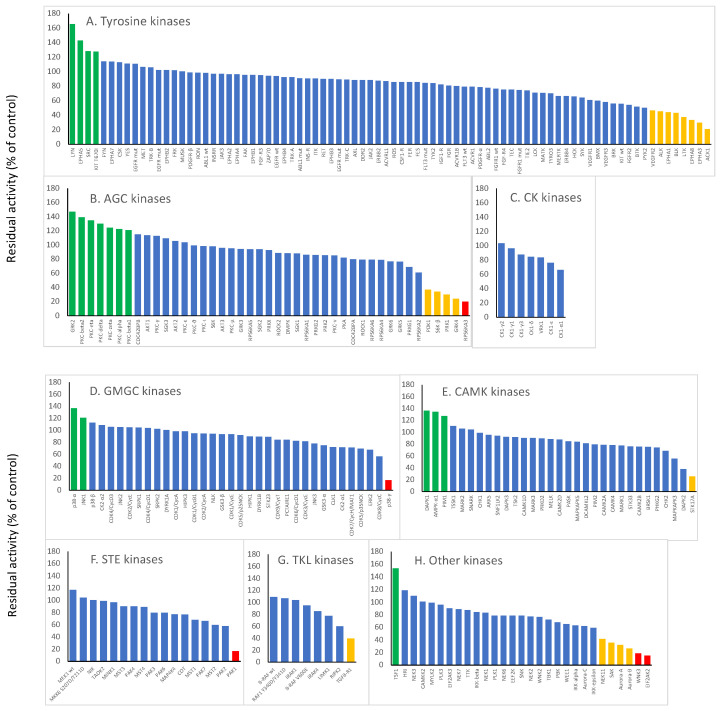
Inhibition of the enzymatic activity of 252 human kinases by CSA. A fixed concentration of 10 µM CSA was used for screening. Residual activities of <20% compared to untreated control (=100%) were considered as strong inhibition of enzyme activity (marked in red), while residual activities in a range of 21–50% were considered as weak inhibitory activities (marked in orange). Increased activities (>120%) upon CSA treatment compared to untreated control were evaluated as stimulation of enzyme activity (marked in green). All other results indicated no effect or weak effects (marked in blue).

The enzymatic activity profiling of 252 kinases from diverse protein kinase classes was performed upon treatment with CSA (10 µM). The results of this kinome activity investigation are displayed in Figure 1, according to the classes they belong to, i.e., tyrosine kinases (TKs), AGC kinases (protein kinases A, G, and C), CMGC kinases (CDK, MAPK, GSK3, and CLK families), calmodulin/calcium-regulated kinases (CAMK), casein kinases (CK), STE kinases (homologs of yeast Ste7, Ste11, and Ste20), and other kinases that do not fit in one of the aforementioned classes.

It was obvious that CSA did not inhibit kinase activity in a monospecific manner, i.e., several rather than single kinases were inhibited by this compound. Five kinases were strongly inhibited by CSA, with a residual enzymatic activity below 20% compared to untreated controls: RPS6KA3 (AGC kinase), PAK1 (STE kinase), p38γ (GMGC kinase), WNK3 (serine/threonine-protein kinase WNK3), and EIF2AK2 (other kinases). These results for these kinases are marked as red bars in Figure 1. Several enzymes from all kinase families were intermediately inhibited (with a range from 21 to 50% residual activity compared to untreated control). These results for these kinases are marked as orange bars in Figure 1. Interestingly, we found not only inhibitory effects of CSA but in some cases also stimulation of enzymatic activity. The results of kinases whose enzymatic activity was activated upon CSA treatment are marked in green (Figure 1). All other kinases revealed weak or no effects after treatment with CSA (with a range from 51 to 120% activity compared to untreated control, blue bars in Figure 1).

**Figure 2:**
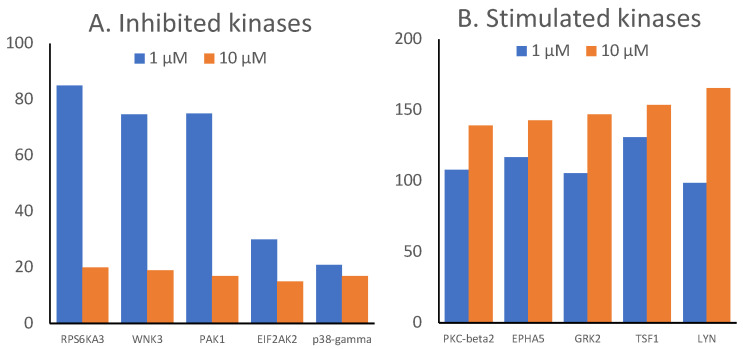
Dose-dependent inhibition and stimulation of human kinases by CSA. Concentrations of 1 and 10 µM were used to measure the effect of CSA on each of the top 5 inhibited and the top 5 stimulated kinases from Figure 1.

We exemplarily verified the results of the most inhibited and the most stimulated kinases by comparing the dose-dependent inhibitory effects of two different concentrations (1 and 10 µM). Figure 2A displays the dose-dependent inhibition of kinase activities, while Figure 2B shows dose-dependent activation of selected kinases.

### 2.2. Molecular Docking Studies

Since we aimed to identify kinases as potential targets of CSA to explain its anticancer activity, we focused on those kinases which were most inhibited by CSA (i.e., EIF2AK2, p38γ, PAK1, RPS6KA3, and WNK3) as well as those, which were most stimulated by this compound (i.e., EPHA5, GRK2, LYN, PRKCB, and TSF1). Therefore, we were interested to investigate whether the inhibition and stimulation of enzymatic activity might be due to the binding of CSA to these proteins. For this reason, we selected the three-dimensional structures of these 10 proteins from the Protein Data Bank and performed molecular docking in silico to assess the interaction of CSA with these proteins.

**Figure 3:**
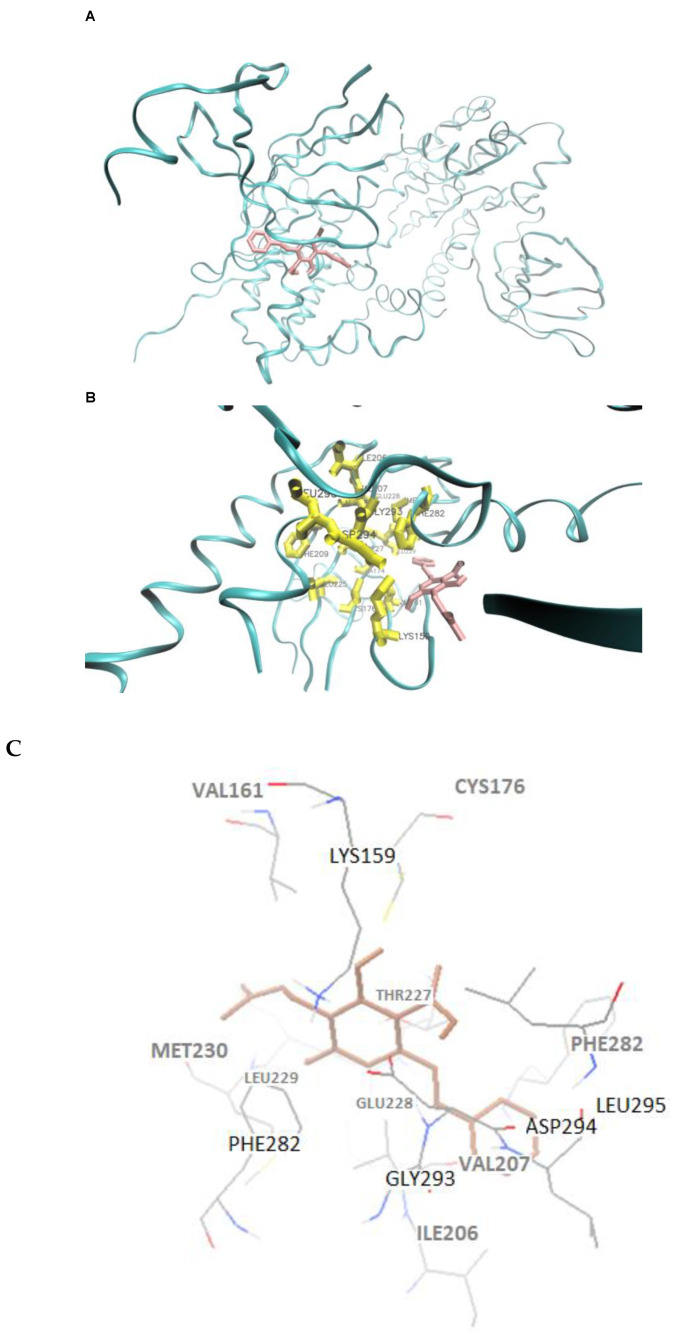
Molecular docking of CSA to WNK3. (**A**) Docking of CSA (pink) to the WNK3 binding site (PDB ID: 5o2b). (**B**) Visual representation of CSA interactions with the amino acids (yellow) in the binding pocket of WNK3. Visual Molecular Dynamics 1.9.4 (VMD) was used for docking visualizations. (**C**) 2D representation of the CSA interactions with WNK3.

As an initial step, we performed a blind docking approach to see whether there might be interactions between CSA and these 10 proteins. The binding energies ranged from −7.8 to −5.33 kcal/mol (LBE) and from −6.93 to −4.80 kcal/mol (MBE). The pKi values ranged from 1.81 to 127.29 µM (Table 1). These blind docking results clearly revealed that the WNK3-CSA interaction exhibited the best affinity among all 10 targets. Therefore, we performed defined docking with WNK3 and CSA. For this reason, a grid map was generated that covered the CSA-binding domain of WNK3 in the blind docking approach. Using defined docking, even better binding affinities (LBE: −9.65 kcal/mol; MBE: −8.84 kcal/mol) and inhibition constant (pKi: 0.084 µM) were calculated (Table 1), confirming the data obtained from blind docking. The amino acid residues of WNK3 forming hydrophobic interactions with CSA identified by defined docking were Val161, Cys176, Ile206, Val207, Phe209, Thr227, Glu228, Leu229, Met230, Phe282, Gly293, Asp294, Leu295, and Leu297. Hydrogen bonding was found with Lys159. Figure 3 depicts the binding of CSA to WNK3 and the participating amino acids. These results indicated that the WNK3 binding and the inhibition of its enzymatic activity may be a major mode of action of CSA among this panel of kinases.

As a positive control, we used PP-121, which is a known inhibitor of WNK3 [42]. PP-121 is bound to the same pharmacophore as CSA. As shown in Table 1, the binding affinities of PP-121 were comparable to those of CSA either in blind or defined docking. The predicted binding constants (pKi) were even a little bit higher than that of CSA, indicating that CSA binds and inhibits WNK3 with a similar or even better efficacy than the control compound PP-121.

Then, we compared the amino acid residues involved in PP-121 and CSA binding to WNK3.

It was intriguing to see that 9 of 10 amino acids involved in PP-121 binding to WNK3 were also involved in the CSA–WNK3 interaction. Since the binding of PP-121 to WNK3 was characterized by co-crystallization studies [42], we compared the amino acids determined by co-crystallization and those predicted by defined docking (Table 2). Eight out of 10 amino acids predicted by defined docking were verified by co-crystallization, indicating that the molecular docking approach predicted the binding mode with 80% correctness. Finally, nine amino acids involved in CSA binding (defined docking) were also found in PP-121 binding (either co-crystallization or defined docking) (Table 2). Taken together, this analysis showed that CSA bound to the same pharmacophore as the known WNK3 inhibitor PP-121, and, thus, both compounds shared the same amino acid patterns to a large extent.

### 2.3. Drug Resistance Profiling

After having identified proteins that may serve as targets of CSA, the question arises as to how important these targets might be for cancer therapy. To address this question, we asked whether these targets may play a role in (1) resistance to standard chemotherapy and (2) in the survival of cancer patients.

By addressing the first question, we focused on the five kinases that were inhibited by CSA and investigated whether these five proteins may be correlated with resistance to clinically established standard anticancer drugs. If these kinases are involved in resistance to clinically used drugs, their inhibition by CSA might sensitize cancer cells to standard chemotherapy.

With the second question, we wanted to clarify, whether the expression of these kinases is correlated with a poor prognosis and worse survival times of cancer patients. In this case, inhibition by CSA might improve survival prognosis, which would be another attractive feature for the use CSA in the clinic.

To address the first question, we correlated the microarray-based mRNA expression of the 5 CSA targets (EIF2AK3, MAPK12/p38γ, PAK1, RPS6KA3, and WNK3) in a panel of 60 tumor lines of the NCI, USA, with 83 standard anticancer agents. To focus on drug resistance, we calculated direct correlations, where high mRNA expression correlated with high log_10_IC_50_ values (i.e., more resistant tumor cells). To increase the reliability of the calculations, we decided only to consider the results of at least two microarray experiments with a significance level of *p* < 0.05 and a correlation coefficient of r > 0.3. In cases where more than two experiments fulfilled this requirement for the relationship between a drug and a target, we have chosen those two microarray hybridizations with the lowest *p*-value and highest r-value. The results are shown in Table 3. High WNK3 expression was significantly correlated with high log_10_IC_50_ values for 5-fluorouracil, tamoxifen, and crizotinib. Similarly, significant correlations were found between RPS6KA3 expression and fulvestrant, everolimus, and temsirolimus, as well as between PAK1 expression and doxorubicin, epirubicin, mitoxantrone, bleomycin, anastrozole, temsirolimus, and sirolimus. No significant correlations were found between the expression of EIF2AK3 or MAPK12/p38γ and the responsiveness of the tumor cell lines to any of the 83 anticancer drugs that were investigated. These data indicate that WNK3, RPS6KA3, and PAK1 expressions were associated with low responsiveness (resp. resistance) to several clinically established anticancer drugs.

### 2.4. Survival Analysis

To address the second question posed above, we were also interested to see whether the expression of these five kinases might be associated with the survival times of cancer patients. The idea behind this speculation was that if high expression was related to short survival, the inhibition of these targets by CSA might improve the survival prognosis of affected patients.

**Figure 4:**
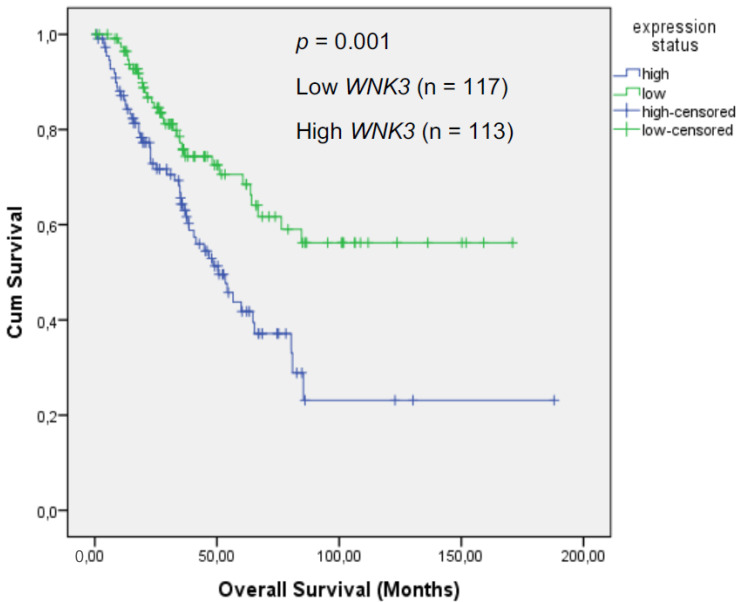
Kaplan–Meier survival analysis of cancer patients, according to their WNK3 expression. Sarcoma patients with high WNK3 mRNA expression had significantly lower survival rates than those with low WNK3 expression (*p* < 0.05). A total of 230 patients (Caucasians) were included in the analysis.

For this reason, patient data belonging to 23 cancer types deposited in the cBioPortal database (https://www.cbioportal.org) were used. To obtain results from homogeneous datasets, only Caucasian patients were included. As analyzed by Kaplan–Meier survival time analysis in a large collection of 230 cases, patients suffering from sarcoma with a high expression of WNK3 died significantly earlier than sarcoma patients with a low WNK3 expression (*p* = 0.001; Figure 4). This result indicates that WNK3 may be a prognostic factor for the overall survival of sarcoma patients. WNK3 expression had no influence on cancer patients affected with other tumor types. Moreover, the expression of none of the other four kinases significantly correlated with the overall survival of cancer patients.

## 3. Discussion

Cajanin stilbene acid (3-hydroxy-4-prenyl-5-methoxystilbene-2-carboxylic acid) is a low abundant natural stilbene isolated from Pigeon Pea (*Cajanus cajan* (L.) Millsp.), a common food crop used in many parts of the world for nutritional purposes. CSA exhibits various biological activities, ranging from antibacterial to neuroprotective effects [40,43,44,45,46]. In our previous investigations, we found that CSA was active in vitro and in vivo against breast cancer with the estrogen receptor and the oncogenic c-MYC as possible targets [39,40,41]. However, CSA is also active in other tumor types where these two targets do not play a role. Therefore, it is evident that other cancer-relevant targets have to be involved in the molecular modes of action of CSA. Kinases are generally accepted as important players in tumor development and progression and are, hence, important targets for the development of tumors [34]. For this reason, we were interested to investigate the human kinome to identify novel potential targets of CSA.

As expected, CSA did not mono-specifically inhibit one single kinase but a couple of kinases, each to a different extent. On the other side, we did not observe a broad-spectrum inhibitory activity across the kinome-wide testing, indicating that specificity, at least to some extent, was visible. Multi- rather than mono-specificity is a typical and usual characteristic of many, if not all, natural products [38]. This is a strategy of plants and microorganisms that was successful during the evolution of life. An organism without a cellular immune system (such as animals or humans) utilizes chemicals as defense weapons against microbes and herbivores. For this purpose, multi-specific defense chemicals are more effective than mono-specific chemicals [47]. This holds true if such chemical compounds are isolated from plants and used for pharmacological purposes to treat human diseases. Even synthetic compounds are not always monospecific, and it was recognized that the frequent development of resistance to synthetic kinase inhibitors still leaves treatment opportunities open for dual- or multi-specific small molecule kinase inhibitors [48,49,50,51]. Therefore, the multi-target specificity of CSA might be considered as an advantage rather than a disadvantage for cancer therapy. Despite the multi-specificity of CSA, it was possible to identify WNK3 as the main target among the human kinome, which comprised 252 different kinases. WNK3 showed the inhibition of enzymatic activity and the strongest binding affinity towards CSA. This is an important and novel result.

The next question we asked ourselves was, how relevant WNK3 and the other four top inhibited targets (EIF2AK2, p38γ, PAK1, and RPS6KA3) are to cancer. It is not sufficient to identify novel inhibitors for novel targets if these targets are not crucial for cancer development and progression. Assuming that CSA would ever reach the clinical stage for cancer treatment, it would, with all probability, be used as part of a combination chemotherapy, rather than as monotherapy. Therefore, we hypothesized that CSA may bear some potential to overcome resistance to other drugs. Indeed, we found that high expression of WNK3, RPS6KA3, and PAK1 correlated with low cellular responsiveness to some clinically established anticancer drugs. This implies that inhibition of these kinases by CSA may render tumor cells more sensitive to standard chemotherapy. This represents a considerably strong argument to include CSA in existing combination therapy regimens.

The activation of oncogenes and inhibition of tumor suppressor genes lead to tumor growth and progression [52,53]. There is ample evidence that oncogenic kinases and tumor suppressors do not only contribute to drug resistance but also to cancer progression and worse outcomes for patients [54,55,56,57,58]. Thus, we investigated the prognostic value of the five top inhibited kinases for the overall survival time of human tumors. A Kaplan–Meier survival analysis of the tumors deposited in the cBioPortal database revealed that high WNK3 mRNA expression was significantly correlated with shorter survival of sarcoma patients. This implies that inhibition of WNK3 by CSA might contribute to better survival chances of sarcoma patients. This hypothesis warrants further detailed investigations to clarify whether WNK3 may hold a prognostic or therapeutic significance in sarcomas, as well as in carcinomas and hematopoietic malignancies.

A rather unexpected result was that the enzymatic activity of several kinases in our kinome-wide analysis was not inhibited but stimulated by CSA. The phenomenon of cellular stimulation of otherwise cytotoxic compounds itself is not new and has been termed hormesis [59,60]. Hormesis describes a biphasic behavior, where low doses of a toxic compound exert positive, stimulatory effects on an organism, while at high concentrations the effect is reversed, and toxic effects become visible. Hormetic reactions in biology have been discussed in the context of the defense and resilience mechanisms of organisms against a wide array of toxic stimuli [61,62]. This phenomenon has also been described for cancer [63,64]. It is possible that the stimulation of some specific kinases upon CSA treatment is dose-dependent, i.e., at low CSA concentrations, stimulation can be observed, while high concentrations lead to the expected inhibitory effects. Hormetic effects of CSA might be observable in some kinases but not in others. Thereby, CSA might exert specific therapeutic effects, e.g., by inhibiting WNK3 as a specific target, but provoking unwanted adverse effects by stimulating other low-affinity kinase targets. It is known that off-target effects are involved in hormesis [65]. The relevance of this phenomenon for CSA requires further attention in the future.

## 4. Materials and Methods

### 4.1. Kinase Activity Profiling

The kinase activity profile of CSA was detected by a previously reported protocol by ProQinase GmbH/Reaction Biology Europe GmbH (Freiburg, Germany) at concentrations of 1 and 10 μM in 252 wild-type protein kinases. As an initial step, CSA was dissolved as 100× stock solutions in 100% DMSO. The final DMSO volume was fixed as 1% in all reaction cocktails. A radiometric protein kinase assay (^33^PanQinase^®^ Activity Assay, Freiburg, Germany) was conducted to determine the kinase activity of the 252 protein kinases. The kinase assays were carried out in 96-well Flash PlatesTM from Perkin Elmer (Boston, MA, USA) in 50 µL reaction volumes. The reaction cocktail, along with the process and the calculation, was previously described. The distinction between low and high control of each enzyme was considered as 100% activity [66,67].

### 4.2. Molecular Docking

Molecular docking is a computational approach to determining the interactions between ligands and macromolecules. Selected proteins were remarkably inhibited upon CSA treatment. The three-dimensional structures of these proteins were retrieved from the Protein Data Bank (PDB) (https://www.rcsb.org and accessed date: 26 June 2021) EIF2AK2 (PDB ID: 6d3k), p38γ (PDB ID: 1cm8), PAK1 (PDB ID: 4zji), RPS6KA3 (PDB ID: 4nw6), and WNK3 (PDB ID: 5o2b). The structures of the five most stimulated proteins were also retrieved from the Protein Data Bank: EPHA5 (PDB ID: 2r2p), GRK2 (PDB ID: 4mk0), LYN (PDB ID: 5xyl), PRKCB (PDB ID: 2i0e), and TSF1 (PDB ID: 2buj). The 3D molecular structure of CSA was generated by using CORINA classic and its SMILES string from PubChem (PubChem, Bethesda, USA CID: 9819225) [68] and was then converted into a PDB file (https://www.mn-am.com/online_demos/corina_demo). The protein structures were formed by eliminating crystallographic water molecules and repairing the missing atoms. After CSA and proteins were prepared in PDBQT format, molecular docking was conducted using AutoDock 4 and a Lamarckian algorithm, as previously followed by our group [69,70]. In the case of blind docking of CSA to 10 targets, the dimensions of the grid box were set around the whole protein targets so that CSA could freely move and rotate in the docking space. For the defined docking of CSA, a grid box consisted of 56 (X), 50 (Y), and 50 (Z) grid points in the relevant dimensions, separated by a distance of 0.375 °A between each one. We set docking parameters as 250 runs and 2,500,000 energy evaluations for each cycle. Docking was conducted independently thrice for the calculation of mean values and standard deviations of the lowest binding energies, mean binding energies, and predicted inhibition constants indicated in the docking log files (dlg). The Visual Molecular Dynamics (VMD) software was used to visualize the interaction modes of CSA to target proteins.

### 4.3. Microarray-Based mRNA Expression of Kinases in 60 NCI Tumor Cell Lines

The NCI, USA established a database with transcriptome-wide mRNA expression profiles and log_10_IC_50_ values of cytotoxic compounds from 60 tumor cell lines (https://dtp.cancer.gov; accessed date 26 January 2021) [71,72,73]. In the present investigation, we correlated the cytotoxicity of 83 standard anticancer drugs from different drug classes (alkylating agents, platinum derivatives, antimetabolites, taxanes, anthracyclines, epipodophyllotoxins, camptothecin derivatives, antihormones, tyrosine kinase inhibitors, mTOR inhibitors, epigenetic inhibitors, and various others) to the mRNA expression values for selected kinase genes from different microarray platforms (Stanford, Affymetrix oligonucleotide, or cDNA biochips). For the correlation analysis, we used Pearson’s correlation test (WinStat, Kalmia Inc., Cambridge, MA, USA; accessed date 30 January 2021).

### 4.4. Kaplan–Meier Survival Analysis of Kinase mRNA Expression in Human Tumors

Data of cancer patients deposited in the cBioPortal database (https://www.cbioportal.org; accessed date 26 August 2021) were used for the survival analysis. The procedure has been previously described by us in detail [74]. Patient data of 23 different cancer types were used. The overall survival status was labeled with a two-digit code (living = 1 or deceased = 0). The median value of the mRNA expression for each dataset was defined as the threshold value to assign the expression of individual tumors as being “high” or “low”. Expression levels above the median value were defined as “high” and those with expression levels below the median value were defined as “low”. Kaplan–Meier survival analyses [75,76] were performed with these data sets using SPSS (IBM Corp. Released 2015. IBM SPSS Statistics for Windows, Version 23.0. Armonk, NY: IBM Corp.). The statistical significance was calculated by “log-rank” test statistics.

## 5. Conclusions

In conclusion, we found that CSA inhibits several human kinases by using a kinome-wide approach based on the measurement of enzymatic activities. WNK3 was the most promising target of CSA with the strongest enzyme inhibition in vitro and highest binding affinity in molecular docking in silico. The association between WNK3 mRNA expression and cellular responsiveness to several clinically established anticancer drugs and the prognostic value of WNK3 mRNA expression with the survival times of sarcoma patients can be taken as clues that CSA-based inhibition of WNK3 might improve treatment outcomes of cancer patients and that CSA may serve as a valuable supplement to the currently used combination therapy protocols in oncology.

## Figures and Tables

**Table 1:** Binding energies and pKi values of CSA binding to inhibited and stimulated kinases.

Compound	Effect	Target	Lowest Binding Energy (LBE, kcal/mol)	Mean Binding Energy (MBE, kcal/mol)	Predicted Inhibition Constant (pKi, µM)
Defined docking:					
PP-121 (control)	Inhibition	WNK3	−9.42 ± <0.01	−9.39 ± <0.01	0.124 ± 0.14
CSA	Inhibition	WNK3	−9.65 ± 0.02	−8.84 ± 0.04	0.084 ± 0.002
Blind docking:					
PP-121 (control)	Inhibition	WNK3	−7.29 ± 0.13	−6.98 ± 0.22	4.60 ± 1.02
CSA	Inhibition	WNK3	−7.83 ± 0.05	−6.93 ± 0.29	1.81 ± 0.15
CSA	Inhibition	EIF2AK2	−6.79 ± 0.01	−6.41 ± 0.13	10.43 ± 0.13
CSA	Inhibition	p38γ	−6.18 ± 0.17	−5.61 ± 0.20	30.38 ± 8.82
CSA	Inhibition	RPS6KA3	−6.17 ± 0.09	−5.74 ± 0.12	30.05 ± 4.10
CSA	Inhibition	PAK1	−5.82 ± 0.07	−5.60 ± 0.27	53.66 ± 6.44
CSA	Stimulation	PRKCB	−6.47 ± 0.28	−6.17 ± 0.27	19.53 ± 8.97
CSA	Stimulation	LYN	−6.10 ± 0.05	−5.53 ± 0.28	34.15 ± 2.79
CSA	Stimulation	GRK2	−6.07 ± 0.07	−5.56 ± 0.22	35.68 ± 4.19
CSA	Stimulation	TSF1	−5.75 ± 0.01	−5.16 ± 0.03	61.28 ± 0.81
CSA	Stimulation	EPHA5	−5.33 ± 0.15	−4.80 ± 0.04	127.29 ± 32.74

**Table 2:** Amino acids in the pharmacophore of WNK3 binding to CSA and PP-121.

Compound	Method	No. of Amino Acids	Amino Acids Involved in Binding	No. of Shared Amino Acids
PP-121	Co-crystallization	11	**Lys159**, **Val161**, Ala174, Leu225, **Thr227**, **Glu228**, Leu229, **Met230**, **Phe282**, **Asp294**, and Leu297	
PP-121	Defined docking	10	Gly156, Lys159, **Val161**, Val207, **Thr227**, **Glu228**, **Met230**, **Phe282**, **Gly293**, and **Asp294**	8 (PP-121 co-crystalization vs. PP-121 docking)
CSA	Defined docking	15	**Lys 159**, **Val161**, Cys176, Ile206, Val207, Phe209, **Thr227**, **Glu228**, Leu229, **Met230**, **Phe282**, **Gly293**, **Asp294**, Leu295, and Leu297	9 (PP-121 co-crystalization vs. CSA docking), 9 (PP-121 docking vs. CSA docking)

Bold: involved in all three; underlined: involved in two.

**Table 3:** Significant correlations between the microarray-based mRNA expression of putative CSA targets and the responses of 60 NCI tumor cell lines to standard anticancer drugs.

**Gene**	**Drug**		**Microarray 1**	**Microarray 2**
***WNK3***	5-Fluorouracil	Pattern ID	GC86671	GC178208
		*r*-Value	0.32301	0.34587
		*p*-Value	0.00591	0.00339
	Tamoxifen	Pattern ID	GC86671	GC178208
		*r*-Value	0.30004	0.36186
		*p*-Value	0.01047	0.00243
	Crizotinib	Pattern ID	GC44489	GC178208
		*r*-Value	0.33139	0.34646
		*p*-Value	0.00517	0.00359
***RPS6KA3***	Fulvestrant	Pattern ID	GC96136	GC177750
		*r*-Value	0.30735	0.35198
		*p*-Value	0.00946	0.00336
	Everolimus	Pattern ID	GC96137	GC211674
		*r*-Value	0.31989	0.30472
		*p*-Value	0.00675	0.00947
	Temsirolimus	Pattern ID	GC36220	GC233962
		*r*-Value	0.37459	0.22470
		*p*-Value	0.00204	0.04496
***PAK1***	Doxorubicin	Pattern ID	GC211478	GC33567
		*r*-Value	0.35408	0.35215
		*p*-Value	0.00319	0.00311
	Epirubicin	Pattern ID	GC211478	GC33567
		*r*-Value	0.38281	0.38511
		*p*-Value	0.00150	0.00129
	Mitoxantrone	Pattern ID	GC211478	112634
		*r*-Value	0.32416	0.33716
		*p*-Value	0.00652	0.00482
	Bleomycin	Pattern ID	GC211478	112634
		*r*-Value	0.31395	0.30689
		*p*-Value	0.00819	0.00955
	Anastrozol	Pattern ID	GC70856	GC96507
		*r*-Value	0.37190	0.31179
		*p*-Value	0.00425	0.01459
	Temsirolimus	Pattern ID	GC85014	166874
		*r*-Value	0.39783	0.37298
		*p*-Value	0.00099	0.00196
	Sirolimus	Pattern ID	GC85014	GC191365
		*r*-Value	0.33352	0.39398
		*p*-Value	0.00491	0.0010

## Data Availability

Not applicable.
